# A giant complex pelvic cystic lesion misdiagnosed as a seminal vesicle tumor: A rare case report of prostatic cyst

**DOI:** 10.1016/j.eucr.2025.103202

**Published:** 2025-09-08

**Authors:** Jianbin Gu, Cong Liu

**Affiliations:** Department of Urology, The People's Hospital of Leshan Central District, Sichuan, China

**Keywords:** Prostatic cyst, Pelvic cystic lesion, Seminal vesicle tumour

## Abstract

Giant prostatic cysts are a rare condition. This case report describes a 53-year-old male patient who presented with acute urinary retention. Imaging studies revealed a giant cystic mass in the pelvic cavity, with septa and nodules, adjacent to the seminal vesicles and compressing the prostate and bladder. Preoperatively, we considered it to be a seminal vesicle tumour and completely resected the cyst and right seminal vesicle via laparoscopic surgery. However, postoperative histological examination confirmed it to be a benign prostatic cyst. This study provides new clinical reference for preoperative decision-making in cases of giant prostatic cyst.

## 1 Introduction

Giant prostatic cysts are a type of pelvic cystic lesion, accounting for only 5 % of patients with lower urinary tract symptoms.^1^ Clinically, they typically present with non-specific symptoms such as lower urinary tract symptoms, pelvic compression symptoms, and male reproductive symptoms. These symptoms overlap with those of seminal vesicle tumours, making differential diagnosis particularly challenging. In clinical treatment, both prostatic cysts and seminal vesicle tumours can be treated through radical surgical resection. However, misdiagnosing seminal vesicle tumours as prostatic cysts and performing procedures such as needle aspiration or decompression may lead to tumour progression and treatment delays. Therefore, establishing an accurate diagnosis and developing a personalised treatment plan are prerequisites for ensuring treatment efficacy. This case report presents a case of a giant pelvic cystic lesion misdiagnosed as a seminal vesicle tumour, followed by radical resection of the right seminal vesicle and cyst.

## 2 Presentation of case

**Fig. 1:**
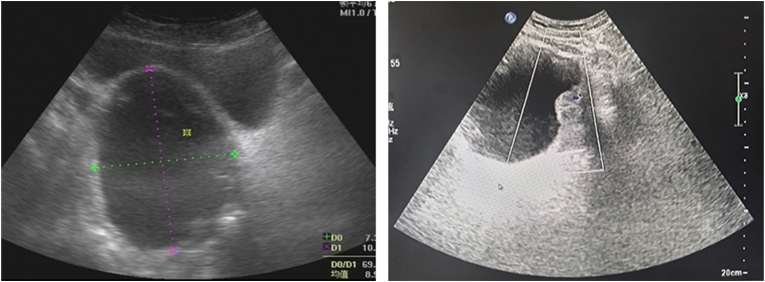
Urological ultrasound: A well-defined, anechoic lesion with clear borders is present posterior to the bladder. High-echoic nodules are visible on the cyst wall and colour Doppler flow imaging (CDFI) shows blood flow signals within the nodules.

**Fig. 2:**
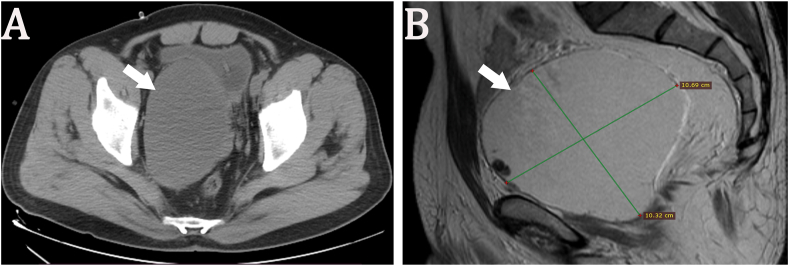
Preoperative imaging: (A) CT showed a cystic mass in the pelvis, compressing the prostate and bladder (arrow). (B) MRI showed a cystic mass measuring approximately 10 × 10 cm (arrow).

**Fig. 3:**
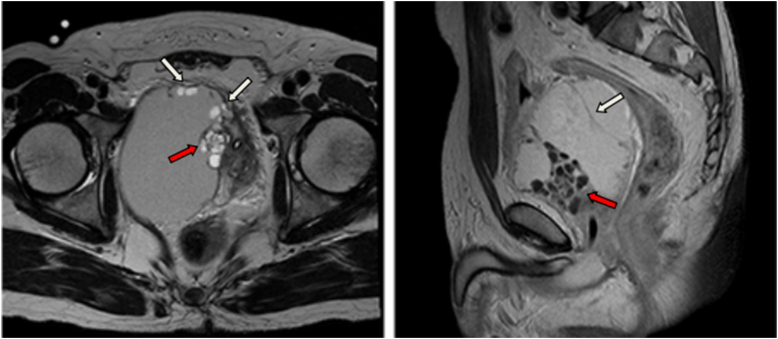
Imaging studies reveal a giant, cystic mass adjacent to the right seminal vesicle (red arrow). The mass has variable density and septa, and there is local thickening of the cyst wall and solid nodules on the anterior walls (white arrow).

We report the case of a 53-year-old previously healthy male patient who presented with 12 months of progressive lower urinary tract symptoms, which evolved from dysuria to drug-refractory acute urinary retention. Accompanying symptoms included decreased ejaculate volume and weak ejaculation, but no storage phase symptoms such as urinary urgency or painful urination. Rectal palpation revealed a prostate of normal size, soft texture, and smooth surface, with no palpable abnormal nodules or tenderness. Imaging evaluation showed structural integrity of the upper urinary tract system without dilative changes. Routine urinalysis, urine culture and urine cytology were unremarkable. Laboratory investigations including biochemical analysis, complete blood count and serum prostate-specific antigen (PSA) assessment were within reference ranges. The patient's International prostate symptom score was 22 and his quality of life score was 5. The patient's urinary system ultrasound showed a well-defined, anechoic lesion with clear borders posterior to the bladder (Fig. 1). MRI and CT scans show a cystic mass in the pelvic cavity adjacent to the right seminal vesicle, measuring approximately 10 × 10 cm in size, with uneven density and internal septa (Fig. 2). The cyst wall is locally thickened, with solid nodules on the left wall and anterior wall (Fig. 3).

**Fig. 4:**
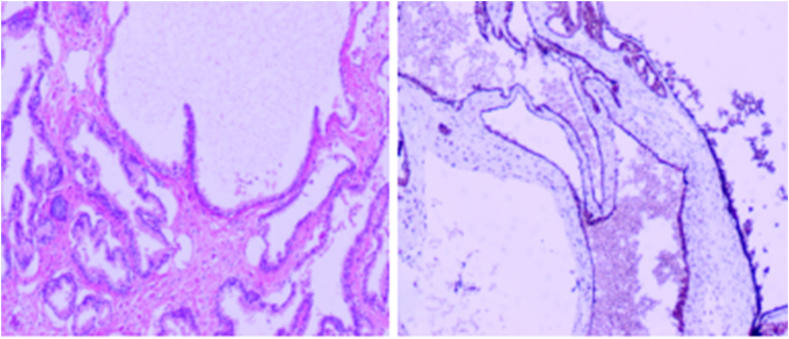
Pathological histological examination revealed that the cyst wall was mainly composed of cuboidal epithelium. The pathological diagnosis was prostatic cyst.

Based on preoperative imaging evaluation, the pelvic cystic mass was highly suspected to be a tumour originating from the seminal vesicles. Given the large tumour size, deep location in the pelvic anatomical space, and difficulty in distinguishing between benign and malignant tumours, a multidisciplinary team decided to perform laparoscopic radical tumour resection combined with right seminal vesicle resection. During the operation, the tumour was found to be difficult to expose, so a windowing decompression procedure was performed, draining approximately 600 mL of brownish-red fluid that was sent for microscopic analysis and cytology. After further dissection, the tumor and right seminal vesicle were isolated and excised. Postoperative microscopic examination of the cyst fluid revealed no spermatozoa. Pathological histological examination revealed that the cyst wall was mainly composed of cuboidal epithelium. Immunohistochemical staining revealed positive expression of AMACR, P63, CK903 and PSA, and negative expression of Calretinin and WT-1. Ki-67 showed focal positive expression. The pathological diagnosis was prostatic cyst, based on the morphological characteristics and immunophenotype (Fig. 4).

**Fig. 5:**
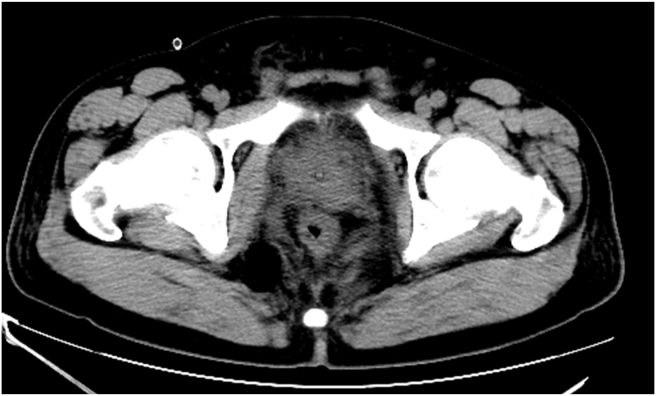
Postoperative imaging evaluation confirmed complete removal of the cyst and complete resolution of lower urinary tract obstruction symptoms.

Postoperative imaging evaluation confirmed that the cyst had been completely removed (Fig. 5). The patient was discharged from the hospital on the fifth day after surgery. After 12 months of follow-up, patient remained stable with complete resolution of LUTS.

## 3 Discussion

Prostatic cysts are a rare benign condition with an extremely low incidence rate. Transrectal ultrasound examinations revealed that only 5 % of patients with symptoms of bladder outlet obstruction or lower urinary tract symptoms had prostatic cyst.^1^ Recent technological developments in the field of ultrasound imaging, coupled with the increasing utilisation of magnetic resonance imaging (MRI), have led to the identification of midline prostatic cysts in a range of 5–14 % of asymptomatic male subjects.^2^ The pathogenesis of prostatic cysts primarily involves congenital factors such as Müllerian duct remnants or developmental anomalies, acquired obstruction resulting from benign prostatic hyperplasia, calculi, or traumatic injury, as well as inflammatory responses including chronic infection or abscess liquefaction.^3^

Most cystic lesions of the prostate do not have specific clinical manifestations, but as the size of the cyst increases, it may exhibit clinical symptoms related to compression of adjacent organs and tissues, such as irritative voiding symptoms, recurrent urinary tract infections, epididymitis, chronic pelvic pain syndrome, haematospermia, low semen volume, or even infertility. Although these symptoms are difficult to distinguish from pelvic cystic lesion, imaging studies provide important evidence for differential diagnosis. Prostatic utricle cysts are typically small, pear-shaped structures located along the midline of the prostate, communicating with the urethra, potentially containing sperm, and not extending superior to the base of the prostate. Müllerian duct cysts, which are often larger than utricle cysts, can be difficult to differentiate radiographically but contain no spermatozoa upon aspiration^4^. Seminal vesicle cysts present as local parenchymal cysts with cyst wall enhancement and water sample density. MRI shows low signal intensity on T1-weighted images and high signal intensity on T2-weighted images.^5^

While the majority of prostatic cystic lesions are benign, it is important to note that prostate cancer can occasionally present as a cystic lesion. The neoplastic incidence of Müllerian duct cysts has been reported to be 3 %. A review of previous cases and relevant literature has resulted in the following characteristics being identified with regard to malignant cystic lesions of the prostate: uneven density or enhanced signal within the cyst; irregular cyst wall with nodules and thickening; multiple cysts or cystic-solid mixed masses, with septa and solid components showing enhanced signal; rapidly growing cysts or recurrent cysts and elevated PSA levels.^6^ These characteristics are similar to the pelvic cystic masses we have reported.

The patient reported in this case had a giant cystic mass in the pelvic cavity. Both preoperative imaging and laboratory investigations suggested a neoplastic lesion of seminal vesicle origin. However, postoperative histopathological examination confirmed it to be a benign prostatic cyst. Upon reviewing the literature, we found that prostatic cysts typically present as single-chambered, thin-walled cystic structures. However, in extremely rare cases, such as those complicated by infection, bleeding, or long-term chronic stimulation, they may exhibit complex cystic features.^7^

For small prostatic cysts that are causing clinical symptoms, cyst puncture and drainage or transurethral cystectomy of the prostate are often recommended. However, the postoperative recurrence rate is relatively high.^8^ In contrast, for giant prostatic cysts, we recommend laparoscopic cystectomy and robot-assisted laparoscopic cystectomy as surgical strategies. These approaches facilitate adequate exposure of the cyst without damaging the surrounding neurovascular bundles or organs, enabling complete resection of the cyst wall. This approach reduces the risk of cyst recurrence and postoperative complications, and promotes faster patient recovery.^9^

In summary, this case report illustrates a rare giant prostatic cyst with imaging characteristics that closely simulate those of a seminal vesicle tumor. We emphasise the importance of considering prostatic origin in the differential diagnosis of pelvic cystic lesions. This case highlights the diagnostic challenges associated with complex pelvic cysts. It provides valuable clinical reference for the management of similar complex cases. Therefore, establishing standardized diagnostic protocols through multidisciplinary collaboration is essential to guide optimal clinical decision-making for such complex pelvic lesions.

## 4 Conclusion

This study presents a rare clinical case of a giant prostatic cyst that was difficult to distinguish from a seminal vesicle tumours. Unlike typical prostatic cysts, the imaging characteristics of this case were highly similar to those of a seminal vesicle tumour. Postoperative immunohistochemical staining revealed positive expression of AMACR, P63, CK903 and PSA, while Calretinin and WT-1 were negative, and Ki-67 showed focal positive expression. The pathological diagnosis was a benign prostatic cyst. This case highlights the complexity of differential diagnosis in pelvic cystic lesions, suggesting that prostate-derived cysts should be included in the differential diagnosis of lesions in the seminal vesicle region as a matter of routine. In terms of treatment, we recommend laparoscopic or robot-assisted laparoscopic surgery for the complete excision of large symptomatic prostate cysts, as this significantly reduces postoperative recurrence and promotes patient recovery. This study provides valuable diagnostic and therapeutic insights for managing complex pelvic cysts. Further validation and optimisation of treatment protocols require an expanded case sample and prolonged follow-up.

## CRediT authorship contribution statement

**Jianbin Gu:** Writing – original draft, Data curation. **Cong Liu:** Writing – review & editing.

## Informed consent

Written informed consent was obtained from the patient for publication of this case report and accompanying images.

## Ethical approval

All data used in this study were derived from the patient's medical records. This report contains no personal information that could identify the patient; thus, ethical approval was waived.

## Funding

This research did not receive any specific grants from funding agencies in the public, commercial, or not-for-profit sectors.

## Declaration of competing interest

The authors declared no potential conflicts of interest with respect to the research, authorship, and/or publication of this article.
